# Halogen Effect in Dual-Catalysis PhotoATRP

**DOI:** 10.1021/acs.macromol.5c02801

**Published:** 2026-01-23

**Authors:** Halil Ibrahim Coskun, Rushik Radadiya, Gorkem Yilmaz, Krzysztof Matyjaszewski

**Affiliations:** Chemistry Department, 6612 Carnegie Mellon University, Pittsburgh, Pennsylvania 15213, United States

## Abstract

The effect of halogen type in dual-catalyzed photoinduced atom transfer radical polymerization (photoATRP) of methyl acrylate (MA) and methyl methacrylate (MMA) was systematically investigated under green LED irradiation (λ ∼ 527 nm) using rhodamine 6G (RD-6G) as a photocatalyst. Poly­(methyl acrylate) and poly­(methyl methacrylate) with ω-bromo and ω-chloro chain ends were synthesized via CuX_2_/ligand (X = Br, Cl) complexes with excess ligand as an electron donor. Kinetic analyses revealed that Br-based systems exhibited significantly faster activation and allowed controlled polymerizations at markedly lower copper and photocatalyst loadings than their Cl-based counterparts. MA polymerizations were faster than MMA despite the latter’s larger ATRP equilibrium constants, attributed to the higher propagation rate constant of acrylates and similar rates of reduction of CuX_2_/ligand deactivators. Optimal ligand selection (Me_6_TREN for MA, TPMA for MMA) was important for control of the polymerization rate and low dispersity. Chain-extension experiments confirmed high chain-end fidelity, and temporal control studies demonstrated efficient light-mediated regulation. These findings provide detailed design guidelines for halogen- and monomer-dependent optimization in dual-catalyzed photoATRP.

## Graphical abstract

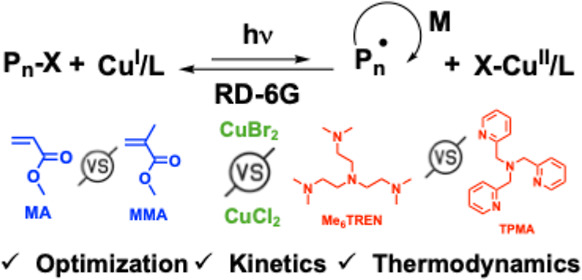

## Introduction

Atom transfer radical polymerization (ATRP)^1,2^ relies on a dynamic equilibrium between radicals and alkyl halides (active and dormant species), which is established by transition metal complexes (e.g., copper, ruthenium, or iron).^3–5^ A lower oxidation state of a metal catalyst (i.e., Cu^I^/L, L: ligand) abstracts the halogen atom (X) from the alkyl halide (P_*n*_-X), in the activation step. This step yields the propagating radicals^6^ (P_n_*), which adds monomer units before being deactivated by the higher oxidation state of catalyst (X–Cu^II^/L) as shown in Scheme 1a. This dynamic equilibrium is in favor of deactivation (*k*_act_ < *k*_deact_), which defines *K*_ATRP_ as shown in eq 1. Therefore, the activity of the catalyst is essential, which is related to the choice of the ligand, the selected halogen, dormant species (monomer), and reaction medium.

1 $$K_{ATRP}=k_{act}/k_{deact}$$

**Scheme 1:**
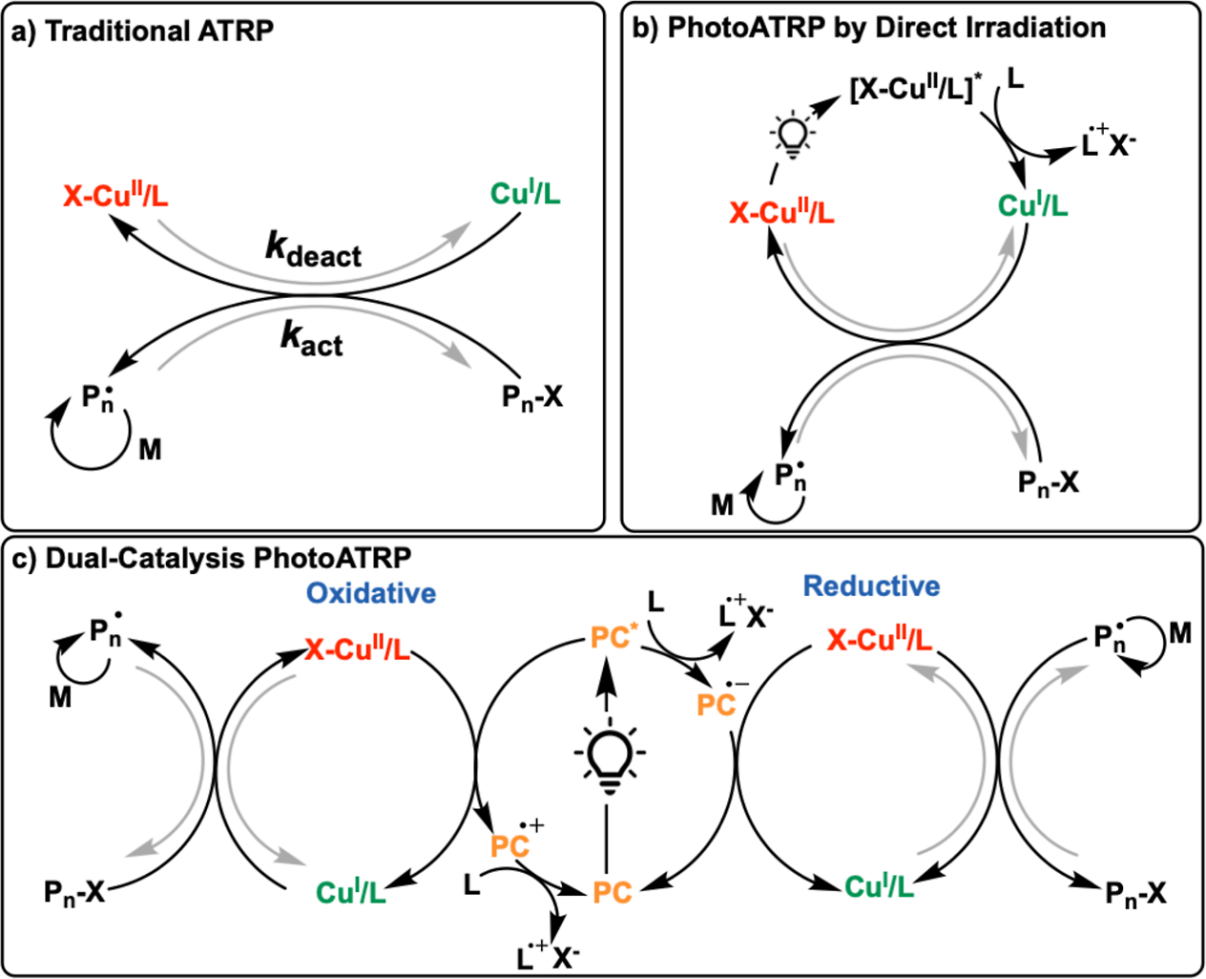
Mechanism of (a) Traditional ATRP, (b) photoATRP by Direct Irradiation, (c) Dual-Catalysis photoATRP

Halogen atom transfer plays a key role in ATRP, as the cleavage of the C–X bond in the activation step depends both on its bond dissociation energy and the halogenophilicity of the catalyst.^7–9^ The dissociation energy of C–X bonds follows the order F > Cl > Br > I.^10,11^ In copper-based systems, the strength of the catalyst-halogen bond follows the same order. A higher halogenophilicity of the catalyst can result in slower deactivation rates, thereby compromising molecular weight control.^12,13^ Therefore, Cl-based ATRP systems usually require higher catalyst loadings and longer reaction times compared to Br-based systems.^14^ Br-end groups are better leaving groups for postpolymerization substitutions, whereas chlorine end groups are more robust due to their stronger C–Cl bond and can provide higher chain-end stability. Particularly, Cl end groups are desired for depolymerization processes, as the more labile bromide groups lead to lactonization at elevated temperatures.^15,16^ Understanding these halogen effects is critical in designing more efficient ATRP systems.^17,18^

Conventional ATRP^19^ required a high catalyst loading to compensate for unavoidable side reactions (i.e., radical termination, oxidation). Subsequently, more active catalysts were developed, allowing control at significantly lower catalyst concentrations.^20,21^ In order to realize a controlled process even at parts per million levels of catalyst concentrations, regenerative ATRP systems have been developed. These methods involve the in situ (re)­generation of the activators from the more stable deactivators. This can be realized by using reducing agents,^22,23^ metallic copper,^24,25^ radical initiators,^26^ electrochemistry,^27^ mechanochemistry,^28^ or photochemical energy.^29–31^

Photochemistry has revitalized polymer science by providing precise spatial and temporal control under mild, ambient conditions.^32–36^ In the earliest photoATRP protocols, ultraviolet irradiation promoted the deactivator complex ([X–Cu^II^/L]*) to an excited state, which was then reduced by sacrificial amines or excess ligand to generate the active Cu^I^/L species (Scheme 1b).^30,37–42^ However, the low penetration and high energy of UV light can limit substrate compatibility and induce side reactions.^43–47^ To overcome these drawbacks, dual-catalyst systems have been developed, in which a photocatalyst (PC) absorbs light and promotes the generation of Cu^I^/L from X–Cu^II^/L, thereby enabling ATRP under a broad spectrum of irradiation wavelengths, including blue, green, red, or NIR LED light.^48–55^ Research has focused on novel PCs that operate at minimal concentrations in organic and aqueous media under ambient conditions while maintaining control over the process. Recently, commercial dyes, including methylene blue,^46^ riboflavin,^56^ eosin Y,^50^ rose bengal,^57^ and rhodamine 6G (RD-6G),^57^ were employed as PCs in dual-catalyzed ATRP systems successfully.

In a dual catalyst system, the generation of the activator can occur via two different pathways, namely, oxidative or reductive quenching mechanisms (Scheme 1c).^58^ The operative mechanism depends on the type of PC. While some PCs predominantly operate on one of the paths, some PCs, such as RD-6G, follow both pathways.^59^ Due to more favorable redox properties, dual-catalyst systems have been predominantly investigated for CuBr_2_/L systems. However, in situations where Cl-chain end functional polymers are desired, the reaction conditions of photoATRP remain unanswered. In addition, a comparison of optimal conditions of photoATRP for acrylate and methacrylate polymer synthesis has not been investigated in detail.

Herein, we report the effect of halogen type on dual-catalyzed ATRP of both methyl acrylate (MA) and methyl methacrylate (MMA). We used RD-6G as PC and green LED irradiation (λ ∼527 nm) in all polymerizations to determine the lowest concentrations of copper catalyst and PC for both systems while preserving control. The structures of all of the materials used are given in Chart 1. Polymerization kinetics, molecular weight evolutions, temporal control, and chain-end functionalities have been studied. Overall conditions for ω-bromo and chloro functional polymethyl acrylate (PMA-Br and PMA-Cl) and poly­(methyl methacrylate) (PMMA-Br and PMMA-Cl) have been studied.

**Chart 1:**
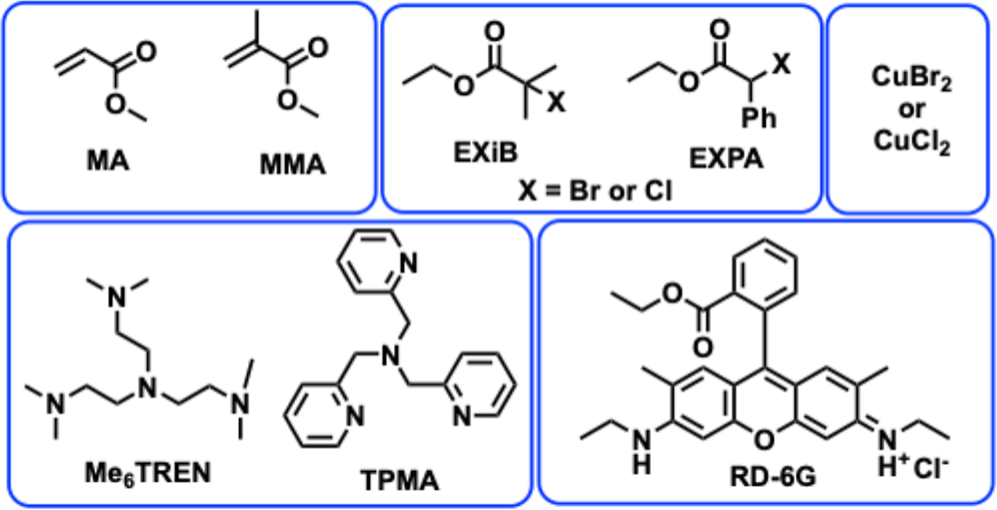
Chemical structures of materials used in this study

## Results and Discussion

### Halogen Effect in PhotoATRP of MA

#### Synthesis of PMA-Br

MA was polymerized in a 50% (v/v) DMSO solution, using ethyl α-bromoisobutyrate (EBiB, 1 mol % targeting DP = 100) as the initiator, RD-6G as the photocatalyst, and Br–Cu^II^/Me_6_TREN as the deactivator, with excess Me_6_TREN as the electron donor (Table 1). Initial experiments were conducted with [CuBr_2_] = 50 ppm, [Me_6_TREN] = 150 ppm, and [RD-6G] = 5 ppm, which resulted in a high conversion (94%) after 2 h and polymers with low dispersity (Entry 1, Đ= 1.12). Controlled polymerizations were achieved even when [RD-6G] concentration was systematically reduced to 0.1 ppm at constant loadings of [CuBr_2_] = 50 ppm and [Me_6_TREN] = 150 ppm (Entry 2 and 3). When [CuBr_2_] loading was reduced to 10 ppm, high conversions with reasonable control could only be attained when a higher excess of Me_6_TREN was employed ([CuBr_2_]/[Me_6_TREN] = 1/15, Đ= 1.37, Entry 4, [CuBr_2_]/[Me_6_TREN] = 1/3, Đ= 1.74, Entry 5). Experiments at [CuBr_2_] = 5 ppm yielded polymers with a broader molecular weight distribution despite the presence of excess Me_6_TREN (Entry 6, Đ= 1.49).

**Table 1:** Results of ATRP of MA with X–Cu^II^/Me_6_TREN and RD-6G^a^

entry	[CuX_2_]/[Me_6_TREN]/[RD-6G]	[CuX_2_]	[CuX_2_] (ppm)	[RD-6G] (ppm)	time (h)	conv. (%)^b^	*M*_n,theo_ (kg·mol^–1^)^c^	*M*_n,app_ (kg·mol^–1^)^d^	D̵^e^
1	0.005/0.015/0.0005	CuBr_2_	50	5	2	94	8.30	8.14	1.12
2	0.005/0.015/0.0001	CuBr_2_	50	1	2	95	8.37	9.30	1.13
3	0.005/0.015/0.00001	CuBr_2_	50	0.1	2	88	8.10	8.40	1.12
4	0.001/0.015/0.0001	CuBr_2_	10	1	2	94	8.30	9.20	1.37
5	0.001/0.003/0.0001	CuBr_2_	10	1	2	46	4.15	4.22	1.74
6	0.0005/0.015/0.0001	CuBr_2_	5	1	2	96	8.45	12.4	1.49
7	0.005/0.015/0.0005	CuCl_2_	50	5	4	90	7.90	11.8	1.54
8	0.02/0.06/0.002	CuCl_2_	200	20	4	81	7.10	10.3	1.44
9	0.04/0.12/0.004	CuCl_2_	400	40	4	70	6.20	7.60	1.50
10	0.04/0.12/0.001	CuCl_2_	400	10	4	93	8.20	8.60	1.32
11	0.04/0.12/0.0005	CuCl_2_	400	5	4	90	7.95	8.65	1.26
12	0.04/0.12/0.0001	CuCl_2_	400	1	4	-	-	-	-

a [MA]/[EBiB or ECiB]/[CuBr_2_ or CuCl_2_]/[Me_6_TREN]/[RD-6G] = 100/1/*x*/*y*/*z*, *V*_MA_ = 1 mL, *V*_MA_/*V*_DMSO_ = 1/1, [MA] = 5.55 M, under Ar (λ ∼527 nm, intensity: 80 mW·cm^–2^).

b Determined by ^1^H NMR.

c Determined by ^1^H NMR *M*_n,theo_ = ([MA]/[Initiator]) × Conv.× *M*_MA_ + *M*_Initiator_.

d Determined by GPC in THF using poly­(methyl methacrylate) standards.

e Đ= *M*_w_/*M*_n_ (by GPC).

#### Synthesis of PMA-Cl

To adapt the protocol to Cl-based systems, EBiB and CuBr_2_ were replaced by ECiB and CuCl_2_, respectively (Table 1). The reactions were carried out for 4 h due to slower activation rates of the Cl-based ATRP (vide infra for kinetic analyses). Experiments with [CuCl_2_] = 50 ppm, [Me_6_TREN] = 150 ppm, and [RD-6G] = 5 ppm resulted in a high conversion (90%), with a broader molecular weight distribution (Entry 7, Đ= 1.54) compared to the Br-based system under identical conditions (Entry 1, Đ= 1.12). Higher [CuCl_2_] loading at a constant [CuCl_2_]/[RD-6G] ratio of 10/1 ([CuCl_2_] = 200 or 400 ppm vs [RD-6G] = 20 or 40 ppm) did not significantly affect the outcome (Entries 8 and 9). Interestingly, when the RD-6G concentration was reduced to 5 and 10 ppm vs a constant [CuCl_2_] = 400 ppm, conversions remained high (>90%), while the control was superior as reflected by the dispersities (Entry 10, Đ= 1.32 and Entry 11, Đ= 1.26). Experiments with 1 ppm of RD-6G did not yield any polymer (Entry 12).

Dual-catalysis ATRP was successfully performed for the synthesis of both PMA-Br and PMA-Cl, although with noticeable differences in reaction conditions and control. The Br-based systems required only very low concentrations of CuBr_2_ (≥10 ppm) and RD-6G (≥0.1 ppm) to reach high monomer conversions (∼90%) and form polymers with low dispersities (Đ≥ 1.12). However, the Cl-based systems required significantly higher catalyst loadings [CuCl_2_] = 400 ppm and [RD-6G] = 5 ppm to give similar conversions, due to the slower activator generation related to the lower reduction potential of the CuCl_2_ catalysts (*E*_1/2_(Cl–Cu^II^/Me_6_TREN) = −0.413 V, *E*_1/2_(Br–Cu^II^/Me_6_TREN) = −0.300 V).^60^ This highlights the Br-based systems as being more favorable under lower catalyst loadings. Furthermore, the control in an ATRP system depends on the *k*_deact_ as shown in eq 2. Thus, higher catalyst loading is needed to compensate for the lower *k*_deact_ values for Cl-based systems to form polymers with low dispersities.^61^

2 $$\mathrm{D̵}=1+\frac{1}{DP}+\frac{\left[RBr\right]k_{p}}{k_{deact}\left[CuX_{2}/L\right]}\times \left(\frac{2}{conv.}-1\right)$$

### Halogen Effect in PhotoATRP of MMA

#### Synthesis of PMMA-Br

Regarding MMA-based systems, polymerizations were performed in a 50% (v/v) DMF solution using ethyl α-bromophenylacetate (EBPA) as the initiator. *K*_ATRP_ for MMA is larger than that of MA systems.^61^ Therefore, TPMA was used instead of Me_6_TREN to slow down the reaction and reach better control.^62^ All experiments for PMMA synthesis were carried out for 24 h (). Initial experiments using [CuBr_2_] = 50 ppm, [TPMA] = 150 ppm, and [RD-6G] = 5 ppm resulted in a 74% conversion, yielding low dispersity polymers (Entry 1, Đ= 1.19). Increasing the TPMA content to 250 ppm ([CuBr_2_]/[TPMA] = 1/5) led to higher conversions (87%), without compromising control (Entry 2, Đ= 1.18). As such, the rest of the reactions were perfTable [Table tbl2]ormed with a [CuBr_2_]/[TPMA] ratio of [CuBr_2_]/[TPMA] = 1/5. When the CuBr_2_ concentration was reduced to 10 ppm, at a constant [CuBr_2_]/[RD-6G] ratio of 10/1, conversion decreased (55%) and control was lost (Entry 3, Đ= 1.64). To reach better control and higher conversion, [RD-6G] was increased to 10 ppm (Entry 4, [CuBr_2_]/[RD-6G] = 1/1, Conv. = 86%, Đ= 1.32). Notably, even at 5 ppm of CuBr_2_ and 5 ppm of RD-6G, the reaction afforded high monomer conversion and yielded polymers with lower dispersity (Entry 5).

**Table 2:** Results of ATRP of MMA with X–Cu^II^/TPMA and RD-6G^a^

entry	[CuBr_2_]/[TPMA]/[RD-6G]	[CuX_2_]	[CuX_2_] (ppm)	[RD-6G] (ppm)	conv. (%)^b^	*M*_n,theo_ (kg·mol^–1^)^c^	*M*_n,app_ (kg·mol^–1^)^d^	D̵^e^
1	0.005/0.015/0.0005	CuBr_2_	50	5	74	7.65	8.30	1.19
2	0.005/0.025/0.0005	CuBr_2_	50	5	84	8.65	9.25	1.18
3	0.001/0.005/0.0001	CuBr_2_	10	1	55	5.75	6.40	1.64
4	0.001/0.005/0.001	CuBr_2_	10	10	86	8.85	9.70	1.32
5	0.0005/0.0025/0.0005	CuBr_2_	5	5	89	9.14	7.9	1.33
6	0.005/0.025/0.0005	CuCl_2_	50	5	14	1.60	5.60	1.54
7	0.02/0.1/0.002	CuCl_2_	200	20	55	5.70	7.00	1.28
8	0.04/0.2/0.004	CuCl_2_	400	40	94	9.60	10.7	1.17
9	0.04/0.2/0.01	CuCl_2_	400	100	93	9.50	10.3	1.18
10	0.04/0.2/0.001	CuCl_2_	400	10	43	4.50	5.40	1.26

a [MMA]/[EBPA or ECPA]/[CuBr_2_ or CuCl_2_]/[TPMA]/[RD-6G] = 100/1/*x*/*y*/*z*, *V*_MMA_ = 1 mL, *V*_MMA_/*V*_DMF_ = 1/1, [MMA] = 4.70 M, under Ar (λ ∼527 nm, intensity: 80 mW·cm^–2^), *t* = 24 h.

b Determined by ^1^H NMR.

c *M*_n,theo_ = ([MMA]/[Initiator]) × Conv. × *M*_MMA_ + *M*_Initiator_.

d Determined by GPC in THF using poly­(methyl methacrylate) standards.

e Đ= *M*_w_/*M*_n_ (by GPC).

#### Synthesis of PMMA-Cl

For PMMA-Cl systems, CuBr_2_ and EBPA were replaced by CuCl_2_ and ECPA, respectively. Initial experiment performed by using [CuCl_2_] = 50 ppm, [TPMA] = 250 ppm, and [RD-6G] = 5 ppm led to a lower MMA conversion (Entry 6, 14%), compared to the Br-based system under identical stoichiometry and [RD-6G]. Similar to the conditions of PMA-Cl, the catalyst concentrations were increased to yield higher conversions due to the lower catalyst activity (Cl–Cu^II^/TPMA vs Br–Cu^II^/TPMA). When [CuCl_2_] was increased to 200 ppm at [CuCl_2_]/[RD-6G] = 10/1, the conversion increased to 55% while yielding low-dispersity PMMA (Entry 7, Đ= 1.28). To achieve higher monomer conversion, [CuCl_2_] was increased to 400 ppm (at a constant [CuCl_2_]/[RD-6G] = 10/1), which resulted in 94% monomer conversion and low dispersity (Entry 8, Đ= 1.17). Increasing the RD-6G concentration did not significantly affect monomer conversion or control (Entry 9). However, decreasing it to 10 ppm resulted in noticeably lower monomer conversion (43%) and slightly higher dispersity (Entry 10).

#### Overall Discussions on Polymerization Conditions

Similar to the MA systems, the Br-based MMA systems provided more efficient polymerizations than their Cl-based analogues, even at significantly lower copper catalyst and photocatalyst concentrations. While 5 ppm of [CuBr_2_] and [RD-6G] were sufficient for Br-based MMA systems, PMMA-Cl synthesis required 400 ppm of [CuCl_2_] and 40 ppm of [RD-6G]. Unlike the Br system, increasing the RD-6G concentration had a minimal effect on the performance, while reducing it led to notable drops in conversion with slight increases in dispersity. This suggests that for the Cl-based system, catalyst concentration plays a more dominant role than the photocatalyst for maintaining control.

Comparing MA and MMA, the generation of radicals (*k*_act_) is faster for MMA polymerization, due to the higher stability of the propagating radicals (3°) and lower bond dissociation energy, compared to radicals in MA polymerization (2°).^18^ Therefore, a less active catalyst system (e.g., X–Cu^II^/TPMA compared to X–Cu^II^/Me_6_TREN) should be used in MMA polymerization, in order to prevent overpopulation of radicals that can lead to termination.^60,62^ In contrast, MMA has a lower propagation rate constant than MA (*k*_p_(MMA) = 270 L·mol^–1^·s^–1^, *k*_p_(MA) = 11,660 L·mol^–1^·s^–1^).^63^ As such, a longer irradiation time was required (see the kinetics).

#### Polymerization Kinetics

To better understand the effect of halogen and the polymer type, the kinetics of polymerization was followed under comparable conditions. Samples were periodically withdrawn from reaction mixtures under argon. Semilogarithmic kinetic plots confirmed the controlled living nature for all polymerizations. Slopes of ln­([*M*]_0_/[*M*]_t_) vs time plots give the apparent rate constants (*k*_p,app_), related to the radical concentration ([R*]) and propagation rate constant as shown in eq 3. In a regenerative ATRP process, *k*_p,app_ depends on the deactivator concentration and its reduction rate constant (*k*_red,app_) as well as the termination rate constant (*k*_t_)­(eq 4). *k*_red,app_ is determined by the structure of the deactivator (X–Cu^II^/L), and thus its reduction potential. Complexes with more negative reduction potentials have lower *k*_red,app_ values (vide infra).

3 $$\ln\left({\left[M\right]}_{0}{/\left[M\right]}_{t}\right)=k_{p}\times \left[R^{*}\right]\times time=k_{p,app}\times time$$

4 $$k_{p,app}=k_{p}\times (k_{red,app}\times {[CuX}_{2}]/k_{t}{)}^{1/2}\times \left[M\right]$$

The X effect on the kinetics of MA polymerization at a molar ratio: [MA]/[EBiB or ECiB]/[CuX_2_]/[Me_6_TREN]/[RD-6G] = 100/1/0.04/0.12/0.0005 ([MA] = 5.55 M in DMSO), was studied. A clear difference in the rates of polymerization was observed, as reflected by the *k*_p,app_ constants; *k*_p,app_ (PMA-Br) = 3.42 × 10^–3^ s^–1^ and *k*_app_ (PMA-Cl) = 2.05 × 10^–4^ s^–1^ (Figure 1a). This is attributed to the more difficult reduction of the Cl-based copper catalyst (*E*_1/2_(Cl–Cu^II^/Me_6_TREN) = −0.413 V, *E*_1/2_(Br–Cu^II^/Me_6_TREN) = −0.300 V)^58–61^ and the higher chlorophilicity of the activators. A similar but less prominent result was observed in the polymerization kinetics of MMA under the following conditions: [MMA]/[EBPA or ECPA]/[CuX_2_]/[TPMA]/[RD-6G] = 100/1/0.02/0.1/0.002 ([MMA] = 4.7 M in DMF). The apparent rate constants were calculated as *k*_p,app_ (PMMA-Br) = 3.25 × 10^–5^ s^–1^ and *k*_p,app_(PMMA-Cl) = 9.80 × 10^–6^ s^–1^ (Figure 1b). These values are closer values compared to Me_6_TREN systems due to a smaller redox potential gap between the TPMA complexes (*E*_1/2_(Br–Cu^II^/TPMA) = −0.245 V, *E*_1/2_(Cl–Cu^II^/TPMA) = −0.325 V), i.e., 80 mV vs 113 mV for Me_6_TREN.

**Figure 1:**
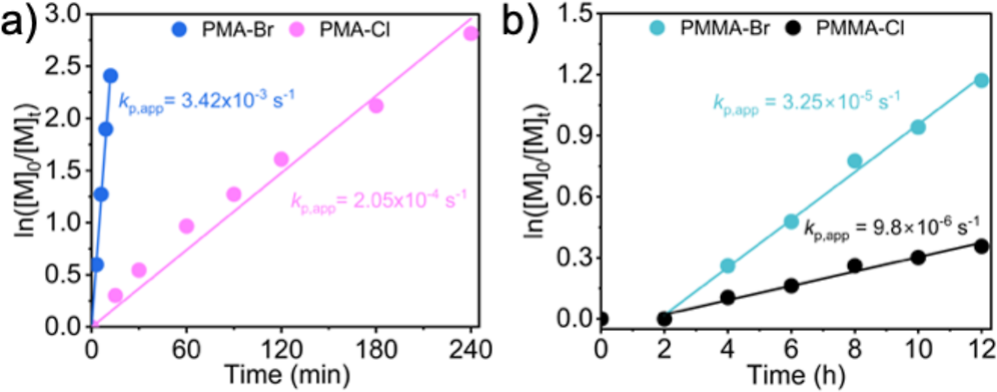
Kinetics of polymerizations at comparable conditions, under Ar, λ ∼527 nm, intensity: 80 mW·cm^–2^: (a) [MA]/[EBiB or ECiB]/[CuBr_2_ or CuCl_2_]/[Me_6_TREN]/[RD-6G] = 100/1/0.04/0.12/0.0005, in DMSO, [MA] = 5.55 M and (b) [MMA]/[EBPA or ECPA]/[CuBr_2_ or CuCl_2_]/[TPMA]/[RD-6G] = 100/1/0.02/0.1/0.002 in DMF, [MMA] = 4.70 M.

When the kinetics of MMA and MA polymerizations were compared for a Br-based system under identical conditions ([MA or MMA]/[EBiB or EBPA]/[CuBr_2_]/[TPMA]/[RD-6G]) = 100/1/0.005/0.015/0.0005, [*M*] = 5.55 M in DMF), a faster polymerization was observed for MA (Figure 2a). Though a higher concentration of radicals was expected in MMA polymerization (radical stability, vide infra), the faster polymerization can be explained by the ∼40-fold larger propagating rate constant of MA compared to MMA.^63^ However, the observed propagating rate constants differ only by ∼3-fold (*k*_p,app_ (PMMA-Br) = 3.06 × 10^–5^ s^–1^ and *k*_p,app_ (PMA-Br) = 8.88 × 10^–5^ s^–1^). This can be attributed to a higher termination rate constant in the ATRP of MA resulting from the catalytic radical termination (CRT).^64^ The secondary radicals react with activators, form paramagnetic R–Cu^II^ species much faster than tertiary radicals, and promote catalytic radical termination. Indeed, reports showed that the majority (∼90%) of the termination reactions in the ATRP of acrylates originated from CRT.^64^ A similar observation was found for Cl-based systems, as shown in Figure 2b. Nevertheless, experimental molecular weights agreed with the calculated values for all polymerization systems (Figures S1–S6).

**Figure 2:**
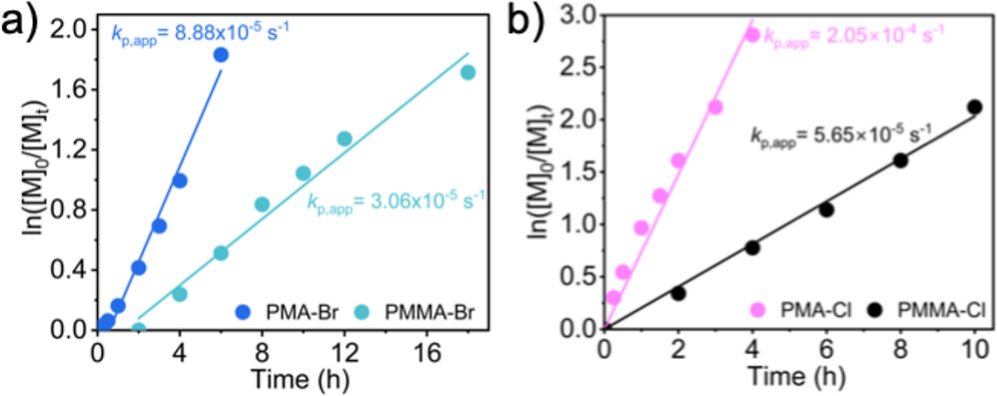
Kinetics of polymerizations at comparable conditions, under Ar, λ ∼527 nm, intensity: 80 mW·cm^–2^: (a) [MA or MMA]/[EBiB or EBPA]/[CuBr_2_]/[TPMA]/[RD-6G] = 100/1/0.005/0.015/0.0005 in DMF, [*M*] = 5.55 M and (b) [MA or MMA]/[ECiB or ECPA]/[CuCl_2_]/[Me_6_TREN]/[RD-6G] = 100/1/0.04/0.12/0.0005 in DMSO, [*M*] = 5.55 M.

To gain a better understanding of the kinetics of polymerization and the relative reduction rates of various X–Cu^II^/L complexes, we conducted UV–vis kinetic studies without monomer (Figures S7–S14). This model system isolates the photoreduction step, specifically the regeneration of Cu^I^/L from X–Cu^II^/L by photoexcited RD-6G and excess ligand, from the more complex ATRP equilibrium during polymerization.^65^ Although these experiments do not capture all aspects of the polymerization system, such as the presence of monomer, dormant chain ends, propagating radicals, or changes in medium polarity, they offer a comparison of the intrinsic photoreduction kinetics across catalytic systems.^13,66^ The broad absorption peaks of the deactivators around 900–960 nm were followed upon irradiation, and the reduction rate constants were determined (Figures S7–14) using the following equations:

5 $$\ln\left(A_{0}{/A}_{t}\right)=k_{red}\times \left[RA\right]\times t$$

6 $$\ln\left(A_{0}{/A}_{t}\right)=k_{red,model}\times t$$

The reduction rate constants from these studies (*k*_red,model_) correlate well with the observed polymerization rate constants (*k*_p,app_), indicating that the photoreduction step significantly influences the overall polymerization kinetics. However, we note that the quantitative values of *k*_red,model_ may differ from the effective reduction rate constants (*k*_red,app_) during polymerization, as described in eq 4, due to different experimental conditions and the influence of ATRP equilibrium. The thermodynamic calculations for the redox processes and their corresponding values are given in Table S1.

#### Chain Extension, Temporal Control, and Varying Degree of Polymerizations

Chain-end fidelity of the synthesized polymers was evaluated by chain-extension experiments. The PMA-Br/Cl and PMMA-Br/Cl were synthesized under optimized conditions. Then, these macroinitiators were successfully extended with the MA monomer. GPC analyses displayed clear shifts to higher molecular weights without tailing or any detectable shoulder, confirming high chain-end fidelity (Figure 3a–d).

**Figure 3:**
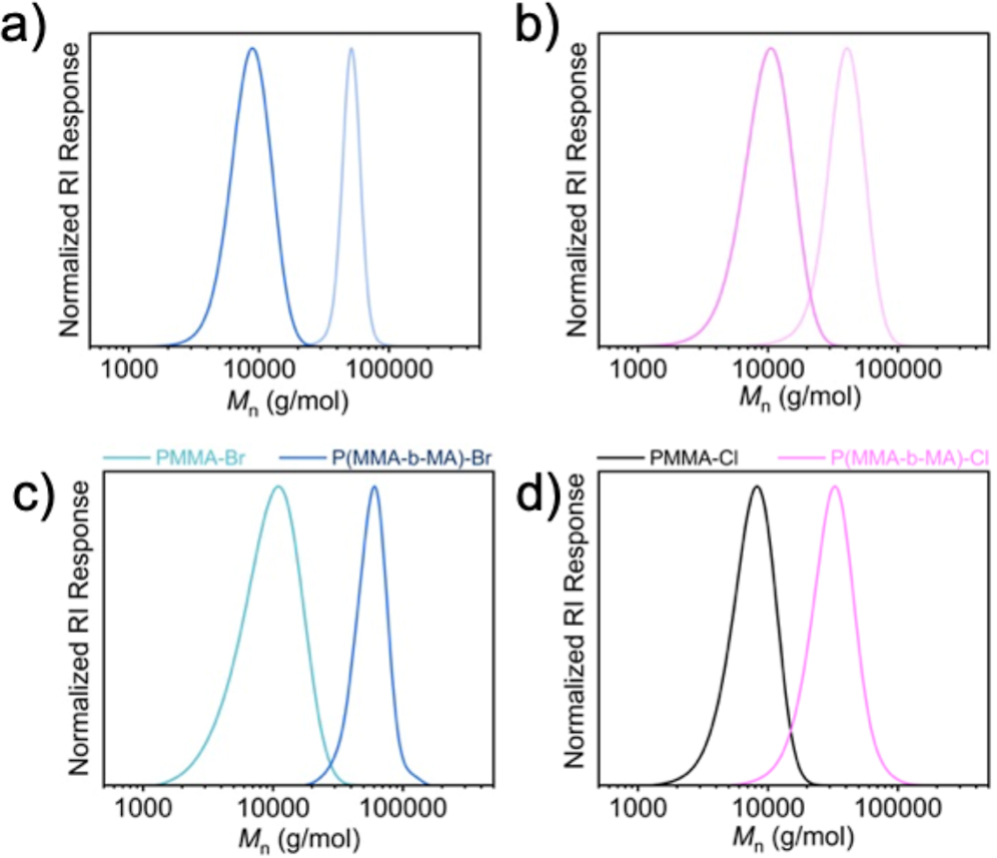
GPC traces of polymers before and after chain extensions. (a) PMA-Br­(pre) [MA]/[EBiB]/[CuBr_2_]/[Me_6_TREN]/[RD-6G] = 100/1/0.005/0.015/0.0001, PMA-Br­(ext) = [MA]/[PMA-Br­(pre)]/[CuBr_2_]/[Me_6_TREN]/[RD-6G] = 500/1/0.005/0.015/0.000, Conv. (%) = 98. (b) PMA-Cl (pre) [MA]/[ECiB]/[CuCl_2_]/[Me_6_TREN]/[RD-6G] = 100/1/0.04/0.12/0.0005, PMA-Cl­(ext) = [MA]/[PMA-Cl­(pre)]/[CuBr_2_]/[Me_6_TREN]/[RD-6G] = 500/1/0.04/0.12/0.0005 Conv. (%) = 65. (c) PMMA-Br (pre) [MMA]/[EBPA]/[CuBr_2_]/[TPMA]/[RD-6G] = 100/1/0.001/0.05/0.001, P­(MMA-*b*-MA)-Br = [MA]/[PMMA-Br­(pre)]/[CuBr_2_]/[Me_6_TREN]/[RD-6G] = 500/1/0.005/0.015/0.0001, Conv. (%) = 98. (d) PMMA-Cl (pre) [MMA]/[ECPA]/[CuCl_2_]/[TPMA]/[RD-6G] = 100/1/0.04/0.2/0.004, P­(MMA-*b*-MA)-Cl = [MA]/[PMMA-Cl­(pre)]/[CuCl_2_]/[Me_6_TREN]/[RD-6G] = 500/1/0.04/0.12/0.0005, Conv. (%) = 52 (all experiments were performed under Ar, λ ∼527 nm, intensity: 80 mW·cm^–2^).

Temporal control of the dual-catalysis system was examined by applying sequential light-on–off cycles periodically. No polymerization was observed during dark periods for all systems, indicating the importance of light on polymerization (Figure 4a–d).

**Figure 4:**
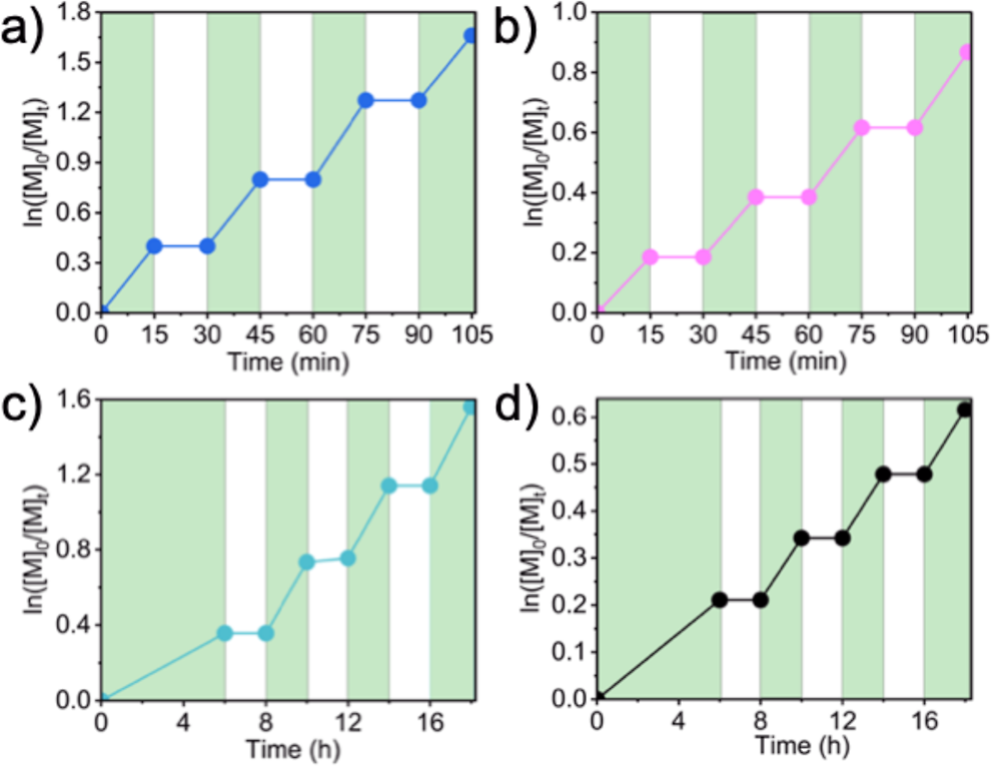
Temporal control results for (a) [MA]/[EBiB]/[CuBr_2_]/[Me_6_TREN]/[RD-6G] = 100/1/0.005/0.015/0.0001. (b) [MA]/[ECiB]/[CuCl_2_]/[Me_6_TREN]/[RD-6G] = 100/1/0.04/0.12/0.0005. (c) [MMA]/[EBPA]/[CuBr_2_]/[TPMA]/[RD-6G] = 100/1/0.001/0.05/0.001. (d) [MMA]/[ECPA]/[CuCl_2_]/[TPMA]/[RD-6G] = 100/1/0.04/0.2/0.004 (all experiments were performed under Ar, λ ∼527 nm, intensity: 80 mW·cm^–2^).

We tested our system for varying targeted degrees of polymerization, ranging from DP = 100 to 800 for all cases. High conversions were reached, and polymers were obtained with low dispersity (Table S2–S3).

## Conclusion

In conclusion, this study demonstrates that the halogen type exerts a pronounced effect on the performance of dual-catalyzed photoATRP of MA and MMA under green light. Br-based systems achieved high conversions and low dispersities at copper catalyst loadings as low as 5–50 ppm and photocatalyst concentrations down to 0.1–5 ppm, whereas Cl-based systems required substantially higher catalyst loadings (up to 400 ppm of CuCl_2_) to reach comparable control. The slower activation in Cl systems arises from the more difficult reduction of CuCl_2_ complexes (i.e., more negative redox potentials). MA polymerizations proceeded faster than MMA under comparable conditions, consistent with the larger propagation rate constant of acrylates. However, MMA required longer irradiation times and higher photocatalyst loadings for optimal control due to slower deactivation. Across all systems, chain-extension experiments verified preserved end-group functionality, and temporal control was achieved via light on/off modulation. Collectively, these results highlight the interplay between halogen type, monomer class, and catalyst/ligand selection, providing a framework for tailoring photoATRP conditions to achieve efficient, well-controlled polymerizations across diverse target structures.

## Supplementary Material



## Notes

Materials, characterization, experimental part, *M*_n_ vs conv. graphs, reduction kinetics of copper complexes by UV analyses, and thermodynamics and kinetic values for different dual-catalytic systems (PDF)
