# Supplementary material for: Halogen Effect in Dual-Catalysis PhotoATRP
Source: Macromolecules. 2026 Jan 23;59(3):1732–9. doi: 10.1021/acs.macromol.5c02801 (PMC12895538; doi:10.1021/acs.macromol.5c02801)
Supplement: Supplementary file 1 [file ma5c02801_si_001.pdf]

Supporting Information

Halogen Effect in Dual-Catalysis PhotoATRP

Halil Ibrahim Coskun[a], Rushik Radadiya[a], Gorkem Yilmaz[a*], Krzysztof Matyjaszewski[a*]

[a]*Department of Chemistry, Carnegie Mellon University, 4400 Fifth Avenue, Pittsburgh, Pennsylvania 15213, United States*

Corresponding Authors: K.M (km3b@andrew.cmu.edu), G.Y. (aliy@andrew.cmu.edu)

Table of Contents

Materials *3*

Instrumentation *3*

Photoreactor for PhotoATRP 3

[1]H Nuclear Magnetic Resonance (^{1}H NMR) 3

Size Exclusion Chromatography (SEC) 3

Spectroscopy 3

Experimental Parts *4*

General procedure for PMA-Br Synthesis 4

General Procedure for PMA-Cl Synthesis 4

General Procedure for PMMA-Br Synthesis 4

General Procedure for PMMA-Cl Synthesis 4

Chain Extension and Block Copolymer Synthesis 5

Temporal Control 5

Supplementary Data *6*

References *23*

Materials

Unless otherwise noted, all chemicals were purchased from commercial sources and used as received. Rhodamine 6G (RD-6G, 99%), copper (II) bromide ($CuBr_2$, 99.99%), copper (II) chloride, ethyl α-bromoisobutyrate (EBiB, 99%), ethyl α-bromophenylacetate (EBPA, 99%), ethyl α-chlorophenyl acetate (ECPA 99%), methyl acrylate (MA, 99%), methyl methacrylate (MMA, ≥99.5%) were purchased from Sigma-Aldrich. Ethyl α-chloroisobutyrate (ECiB) was synthesized as reported previously.[1] Monomers were passed through a column of basic alumina to remove the inhibitor prior to use. Tris(2-pyridylmethyl)amine (TPMA, 99%) and tris[2-(dimethylamino)ethyl]amine (Me_6TREN, 99%) were purchased from Ambeed. Dimethylformamide (DMF, HPLC grade), dimethyl sulfoxide (DMSO, HPLC grade) and tetrahydrofuran (THF, HPLC grade) were purchased from Fisher Chemical. DMSO-d_6 and $CDCl_3$ were purchased from Cambridge Isotope Laboratories, Inc.

Instrumentation

Photoreactor for PhotoATRP

Polymerizations were conducted in 1-drum pressure release vials on a 24-point LED array with green light (527 nm, 80 mW/cm²). The LED array and Lumidox Gen II LED Controller were purchased from *Analytical Sales and Services, Inc.*

^{1}H Nuclear Magnetic Resonance (^{1}H NMR)

^{1}H NMR spectra were recorded on a Bruker Avance III 500 MHz spectrometer with $CDCl_3$ or DMSO-d_6 used as the solvent.

Size Exclusion Chromatography (SEC)

The molecular weights (M_n) and dispersities ($Đ$) were measured relative to poly(methyl methacrylate) (PMMA) standards by gel permeation chromatography (GPC) conducted with an Agilent GPC instrument using THF as the eluent. The GPC was equipped with an RI detector and PSS columns (Styrogel 10^5, 10^3, 10^2 Å) at 35 °C and a flow rate of 1 mL/min.

Spectroscopy

UV-vis spectra were recorded on an Agilent 8453 spectrometer.

Experimental Parts

General procedure for PMA-Br Synthesis

MA (1 mL, 11 mmol), $CuBr_2$ (0.552 μmol, added as 18.7 μL from a stock solution of 29.5 mM in DMSO), Me_6TREN (1.66 μmol, added as 15.7 μL from a stock solution of 105 mM in DMSO), RD-6G (0.0011 μmol, added as 5.18 μL from a stock solution of 0.213 mM in DMSO), and EBiB (21.5 mg, 0.110 mmol) were added. DMSO was added to fill up the volume to 2 mL. The reaction mixtures were transferred to a 1-dram pressure release vial charged under Ar atmosphere. The polymerizations started upon turning on the green LEDs (527 nm, 80 mW·cm^{-2}). Samples were collected with a syringe and analyzed using ^{1}H NMR and GPC techniques. Aliquots were taken at different times under a blanket of Ar to analyze the reaction's kinetics.

General Procedure for PMA-Cl Synthesis

MA (1 mL, 11 mmol), $CuCl_2$ (1.4 μmol, added as 20.2 μL from a stock solution of 68.7 mM in DMSO), Me_6TREN (4.2 μmol, added as 11 μL from a stock solution of 372 mM in DMSO), RD-6G (0.14 μmol, added as 19 μL from a stock solution of 7.4 mM in DMSO), and ECiB (16.6 mg, 0.11 mmol) were added. DMSO was added to fill up the volume to 5 mL. The reaction mixtures were transferred to a 1-dram pressure release vial charged under Ar atmosphere. The polymerizations started upon turning on the green LEDs (527 nm, 80 mW·cm^{-2}). Samples were collected with a syringe and analyzed using ^{1}H NMR and GPC techniques. Aliquots were taken at different times under a blanket of Ar to analyze the reaction's kinetics.

General Procedure for PMMA-Br Synthesis

MMA (1 mL, 9.4 mmol), $CuBr_2$ (0.047 μmol, added as 2.15 μL from a stock solution of 25.6 mM in DMF), TPMA (0.235 μmol, added as 3.23 μL from a stock solution of 72.7 mM in DMF), RD-6G (0.0047 μmol, added as 22.2 μL from a stock solution of 2.11 mM in DMF), and EBPA (22.8 mg, 0.094 mmol) were added. DMF was added to fill up the volume to 2 mL. The reaction mixtures were transferred to a 1-dram pressure release vial under Ar atmosphere. The polymerizations started upon turning on the green LEDs (527 nm, 80 mW·cm^{-2}). Samples were collected with a syringe and analyzed using ^{1}H NMR and GPC techniques. Aliquots were taken at different times under a blanket of Ar to analyze the reaction's kinetics.

General Procedure for PMMA-Cl Synthesis

MMA (1 mL, 9.4 mmol), $CuBr_2$ (3.76 μmol, added as 103.6 μL from a stock solution of 36.30 mM in DMF), TPMA (18.8 μmol, added as 258.7 μL from a stock solution of 72.7 mM in DMF), RD-6G (0.376 μmol, added as 178.3 μL from a stock solution of 2.11 mM in DMF), and ECPA (18.7 mg, 0.094 mmol) were added. DMF was added to fill up the volume to 2 mL. The reaction mixtures were transferred to a 1-dram pressure release vial charged under Ar atmosphere. The polymerizations started upon turning on the green LEDs (527 nm, 80 mW·cm^{-2}). Samples were collected with a syringe and analyzed using ^{1}H NMR and GPC techniques. Aliquots were taken at different times under a blanket of Ar to analyze the reaction's kinetics.

Chain Extension and Block Copolymer Synthesis

Chain extensions were performed using the same procedure as the polymerizations described above. PMA-Br/Cl and PMMA-Br/Cl were synthesized and used as macroinitiators for chain extension and block copolymer synthesis procedures using MA as monomer.

Temporal Control

Temporal control for all systems was investigated with optimal conditions as mentioned above by a sequence of light on/off cycles.

Varying Degree of Polymerizations

Experiments were performed as mentioned in general procedures by adjusting initiator concentration accordingly.

Supplementary Data

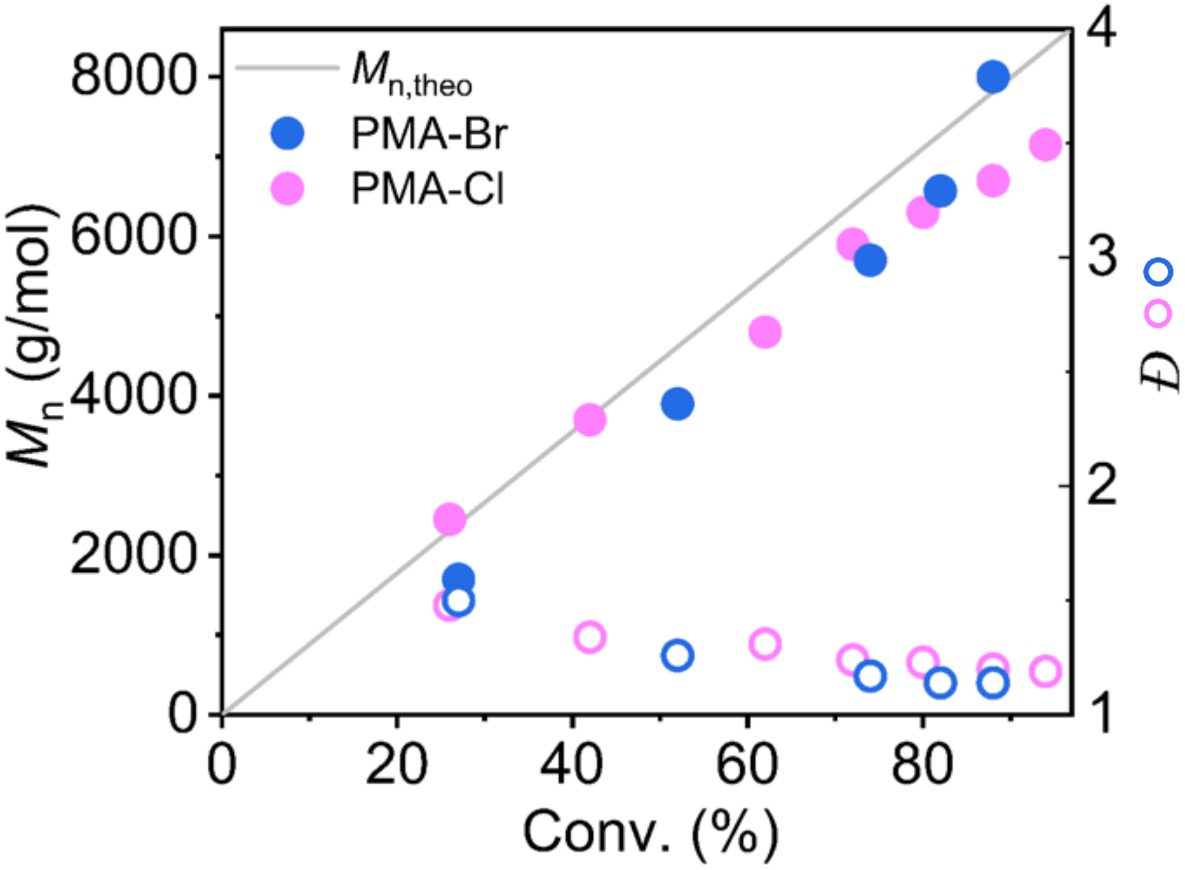

Figure S1. M_n vs conv. plot for Figure 1a.

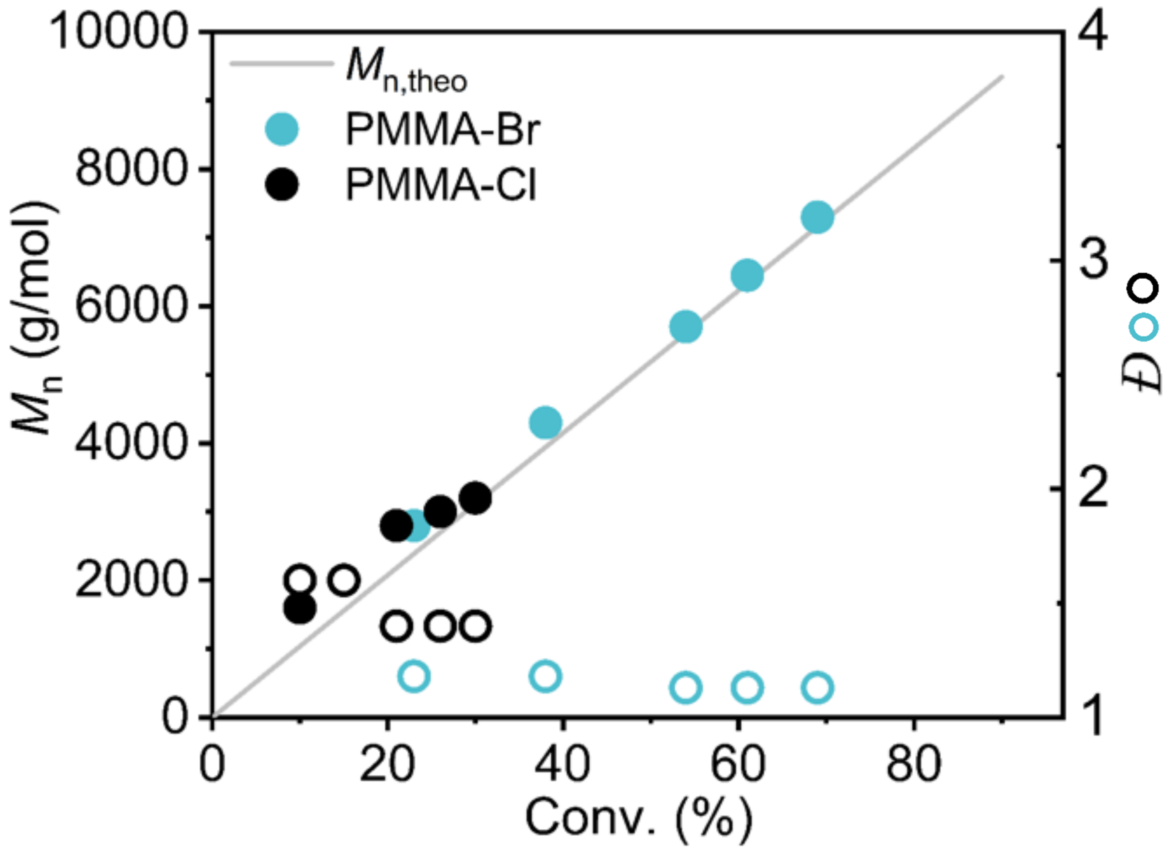

Figure S2. M_n vs conv. plot for Figure 1b.

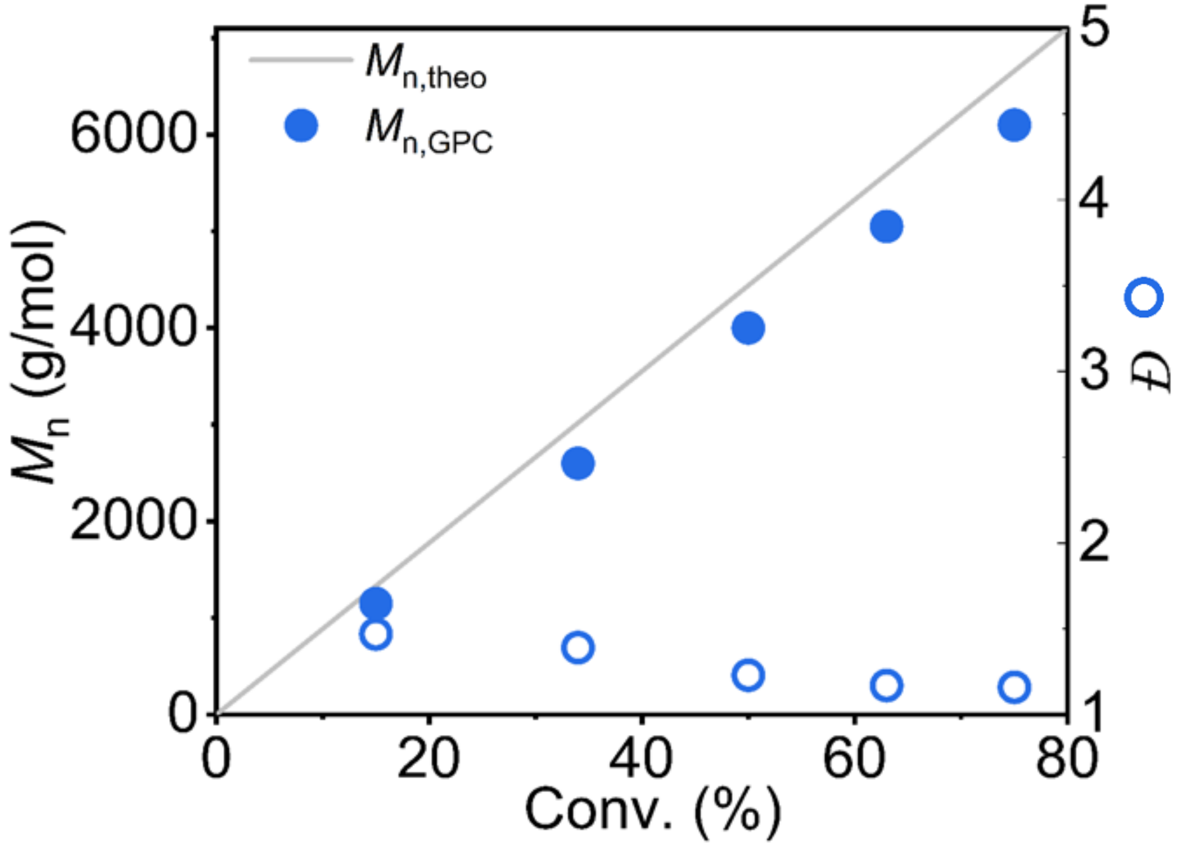

Figure S3. M_n vs conv. plot for PMA-Br synthesis in Figure 2*a*.

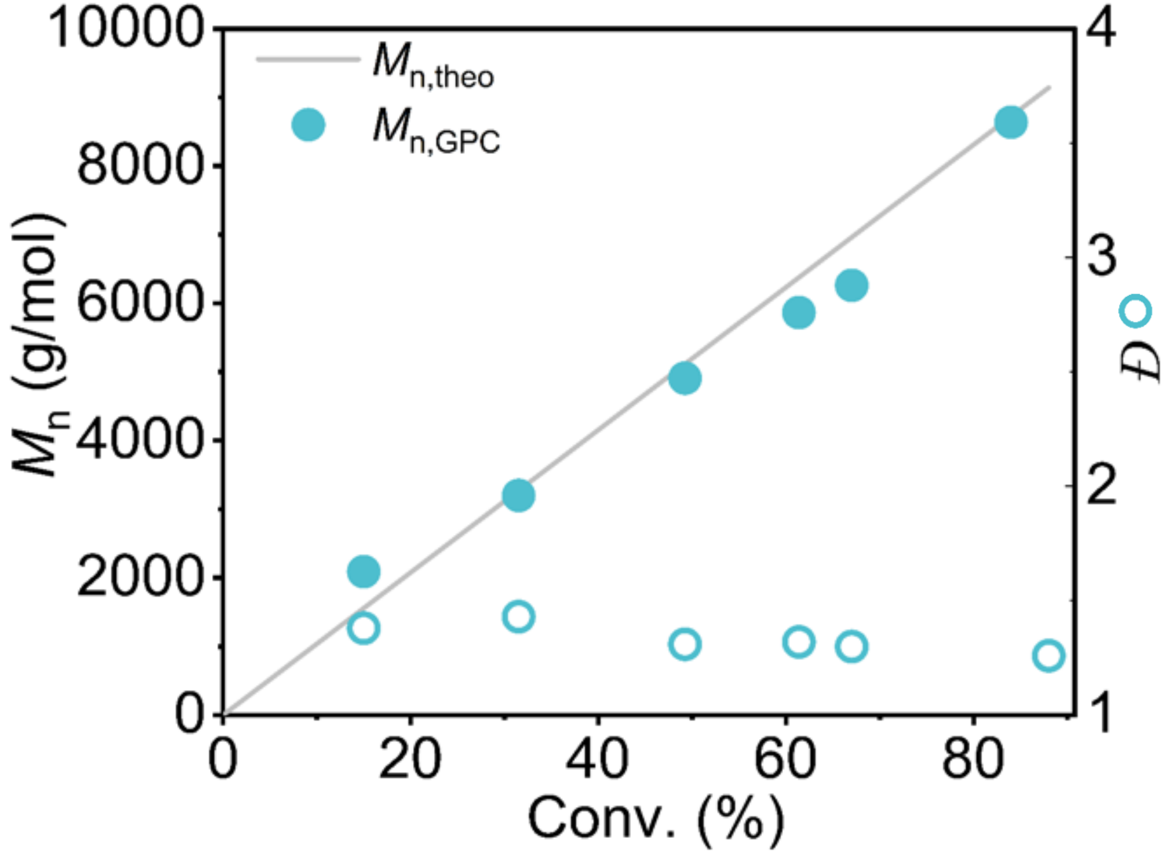

Figure S4. M_n vs conv. plot for PMMA-Br synthesis in Figure 2a.

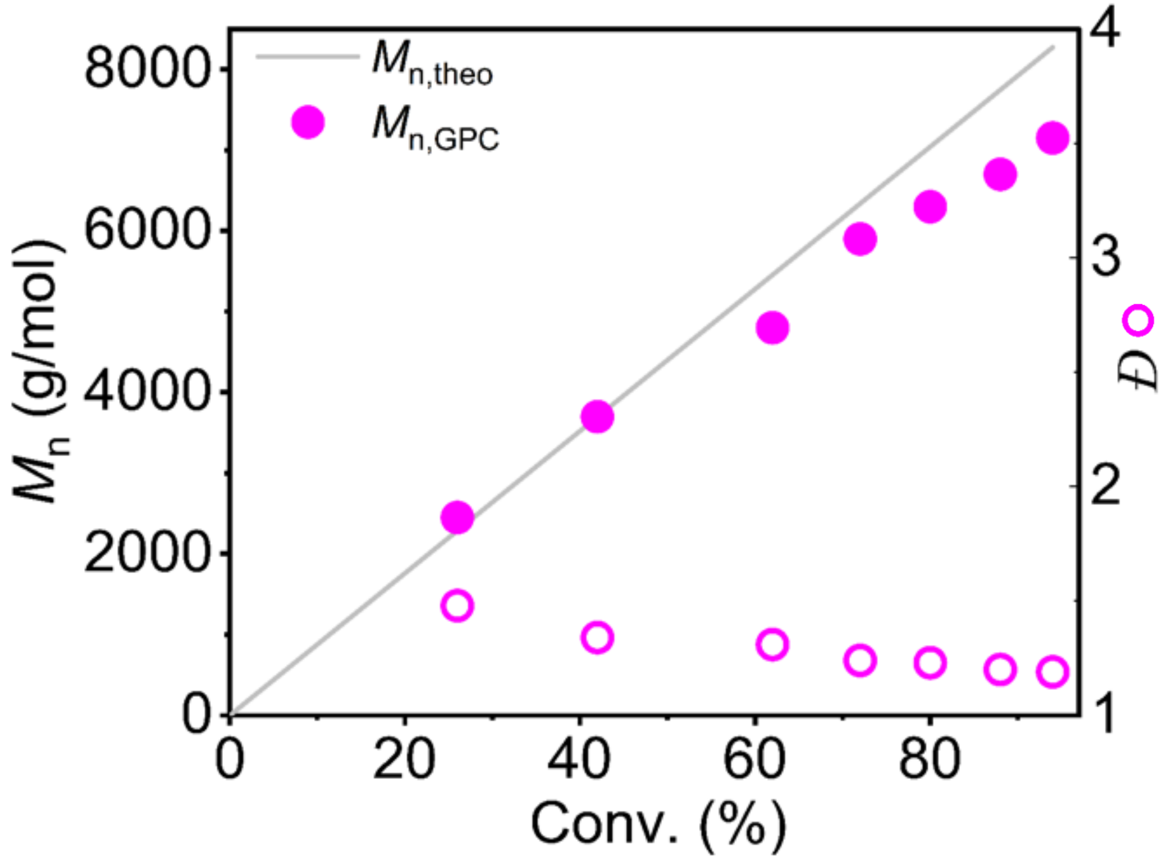

Figure S5. M_n vs conv. plot for PMA-Cl synthesis in Figure 2b.

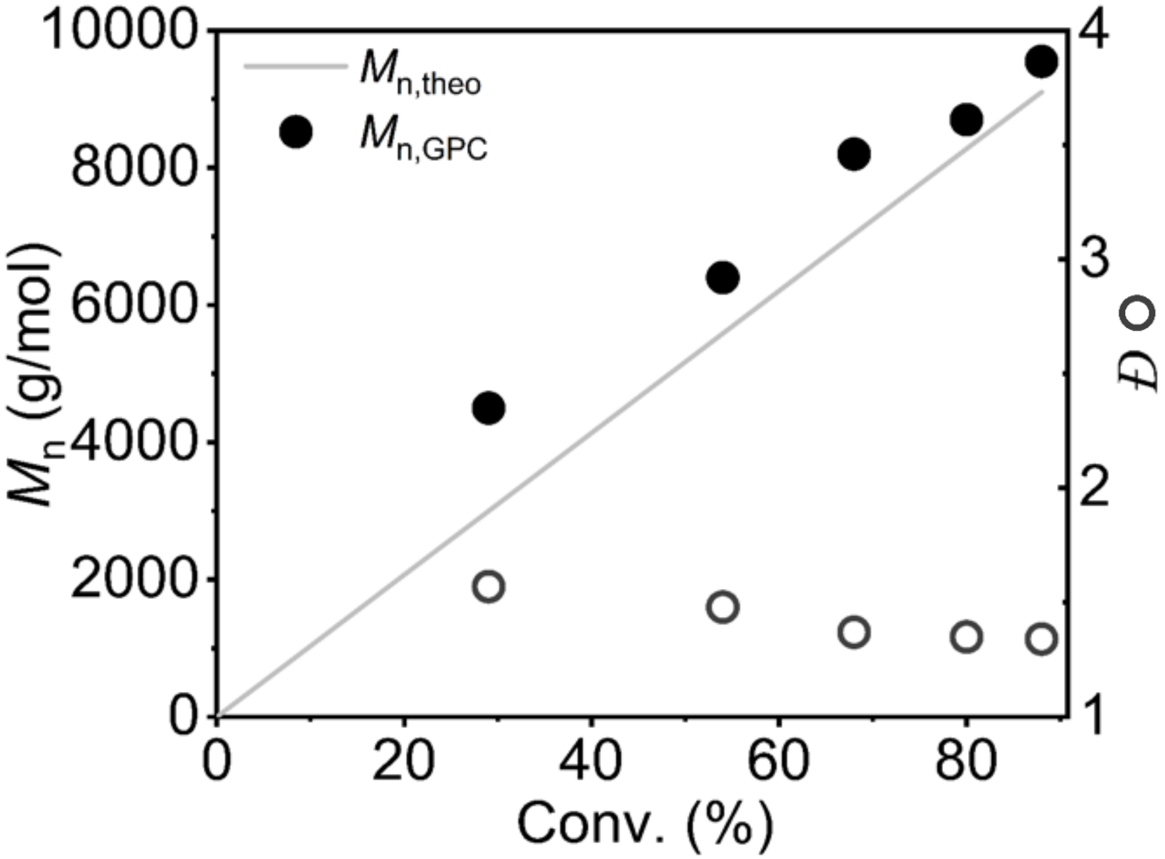

Figure S6. M_n vs conv. plot for PMMA-Cl synthesis in Figure 2b.

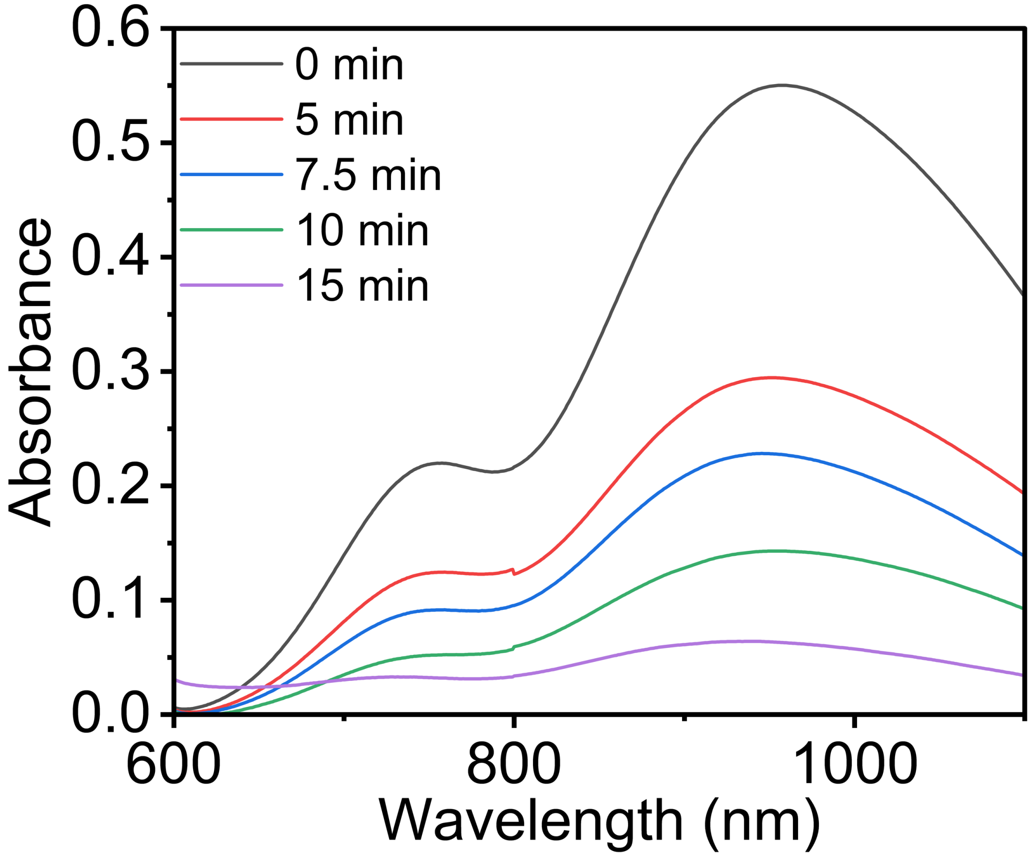

Figure S7. UV-vis analysis of [Br-Cu^{II}/Me_6TREN] in the presence of RD-6G and excess Me_6TREN in DMSO ([$CuBr_2$]/[Me_6TREN]/[RD-6G] = 1/3/0.1, [$CuBr_2$] = 4.55 mM) under light irradiation (525 nm, intensity: 25 mW/cm^2) at different times. This model system experiment was conducted in the absence of monomer to isolate the photoreduction kinetics of the copper complex. It shows the reduction of [Br-Cu^{II}/Me_6TREN] as evidenced by the decrease of the absorption band at ~ 960 nm.

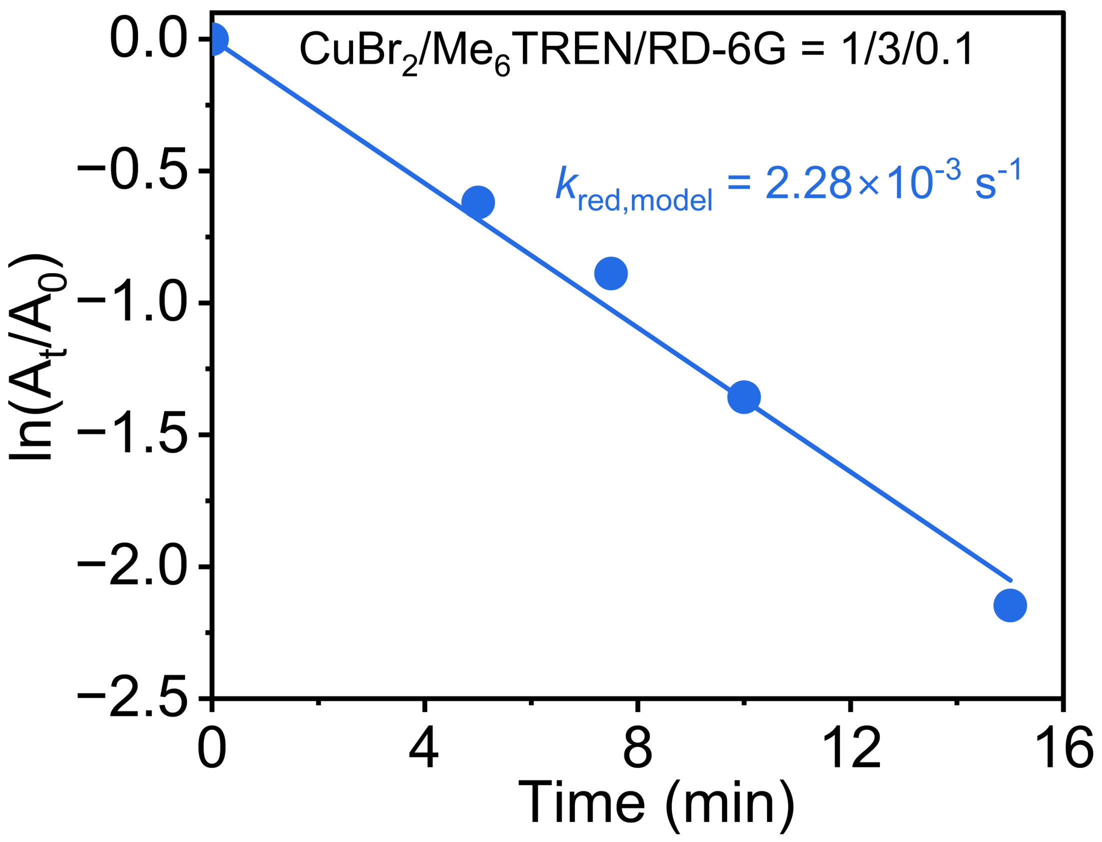

Figure S8. Kinetics of the reduction of [Br-Cu^{II}/Me_6TREN] in the presence of RD-6G and excess Me_6TREN in DMSO. Data is taken from Figure S7.

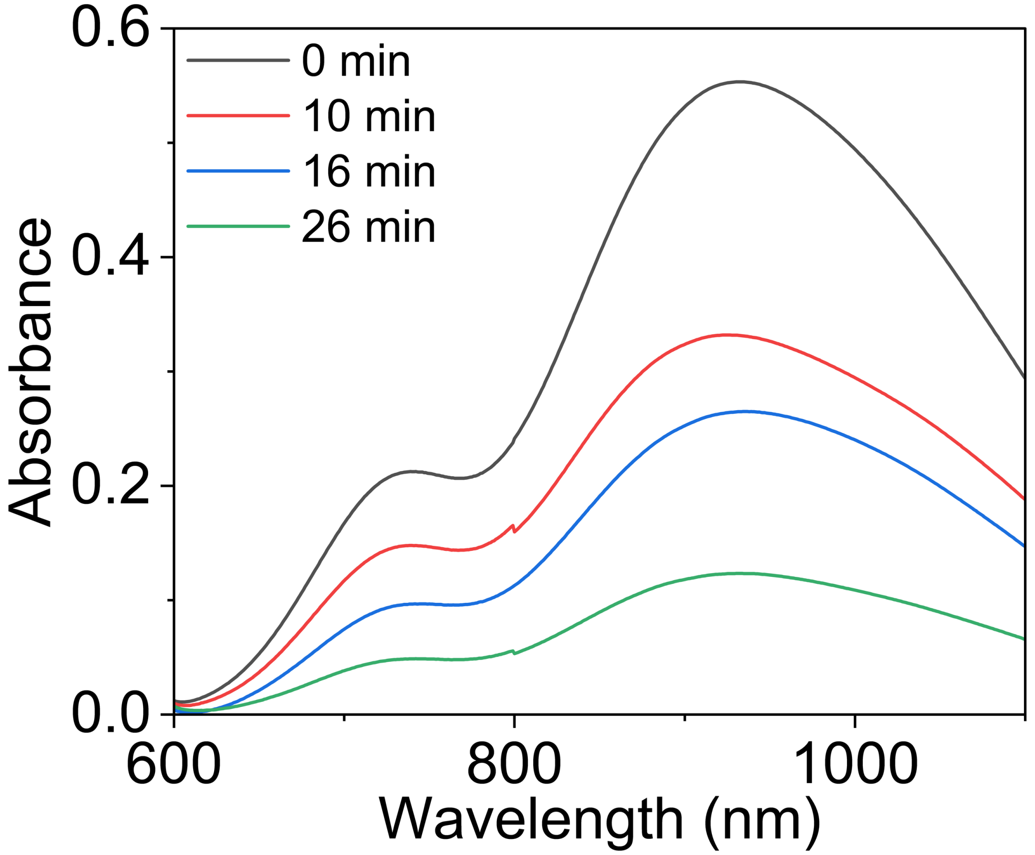

Figure S9. UV-vis analysis of [Cl-Cu^{II}/Me$_6$TREN] in the presence of RD-6G and excess Me$_6$TREN in DMSO ([CuCl$_2$]/[Me$_6$TREN]/[RD-6G] = 1/3/0.1, [CuCl$_2$] = 4.55 mM) under light irradiation (525 nm, intensity: 25 mW/cm^2) at varying times. This model system experiment was conducted in the absence of monomer to isolate the photoreduction kinetics of the copper complex. It shows the reduction of [Cl-Cu^{II}/Me$_6$TREN] as evidenced by the decrease of the absorption band at ~ 935 nm.

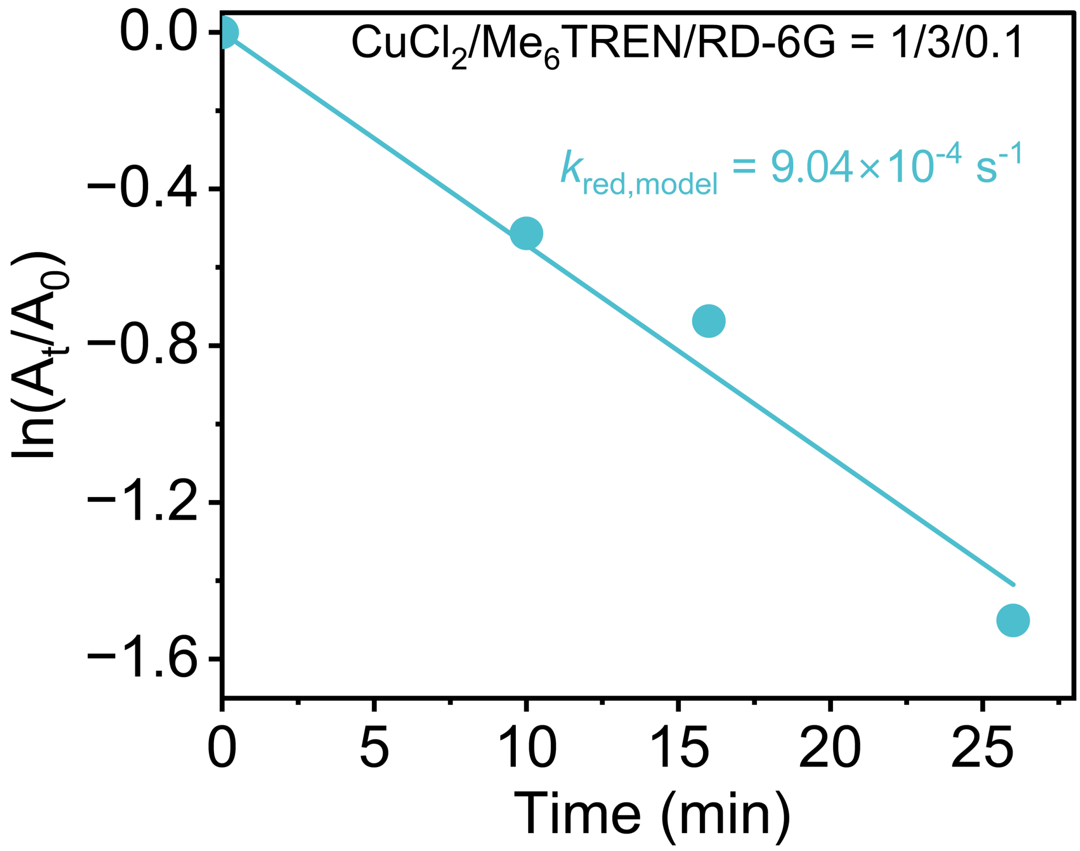

Figure S10. Kinetics of the reduction of [Cl-Cu^{II}/Me_6TREN] in the presence of RD-6G and excess Me_6TREN in DMSO. Data is taken from Figure S9.

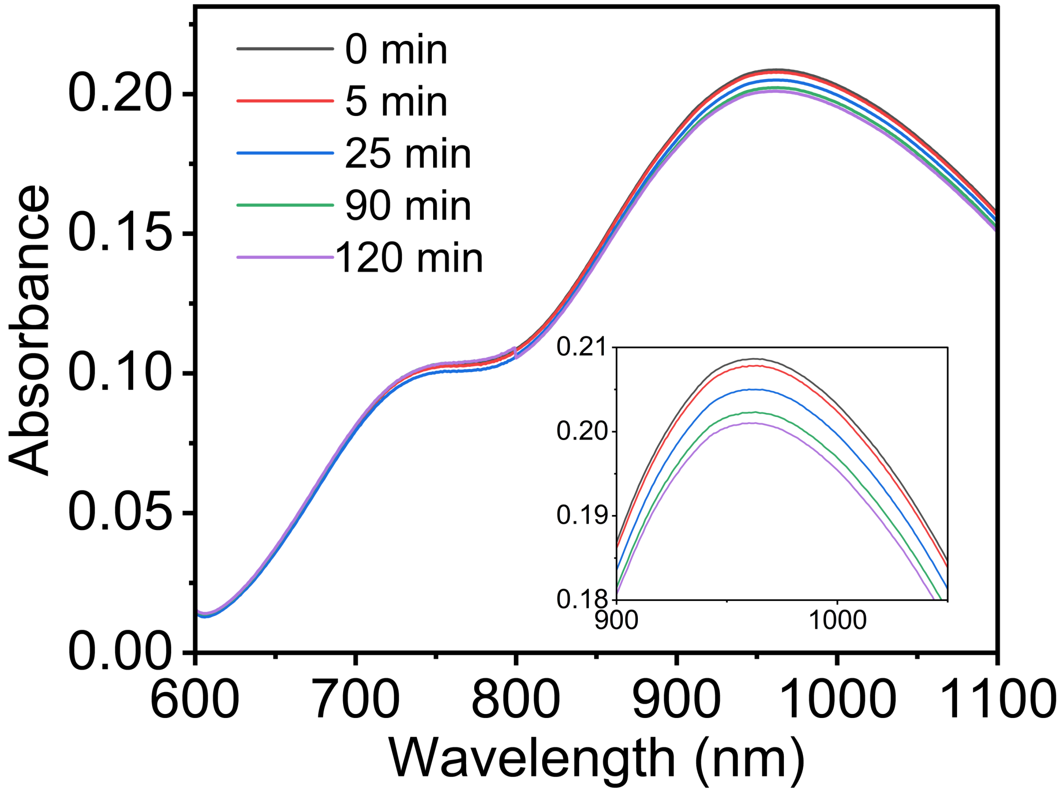

Figure S11. UV-vis analysis of [Br-Cu^{II}/TPMA] in the presence of RD-6G and excess TPMA in DMSO ([$CuBr_2$]/[TPMA]/[RD-6G] = 1/3/0.1, [$CuBr_2$] = 4.55 mM) under light irradiation (525 nm, intensity: 25 mW/cm^2) at varying times. This model system experiment was conducted in the absence of monomer to isolate the photoreduction kinetics of the copper complex. It shows the reduction of [Br-Cu^{II}/TPMA] as evidenced by the decrease of the absorption band at ~ 960 nm.

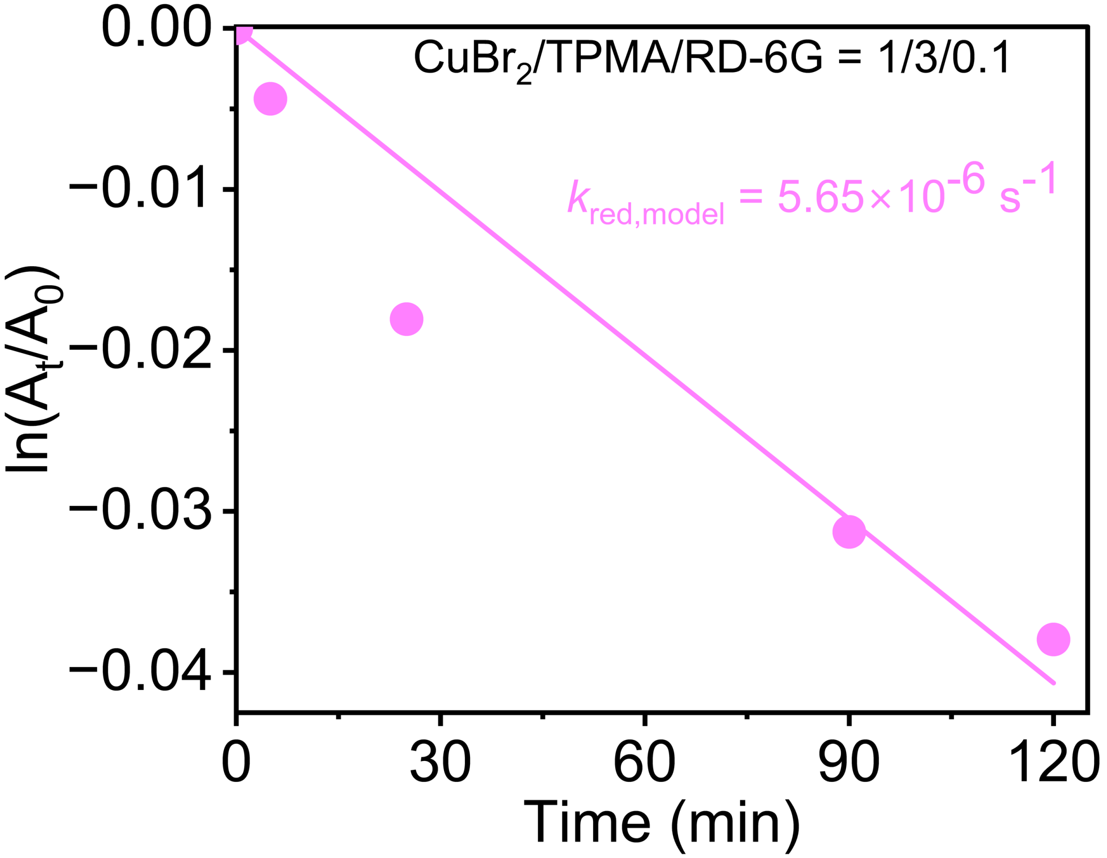

Figure S12. Kinetics of the reduction of [Br-Cu^{II}/TPMA] in the presence of RD-6G and excess TPMA in DMSO. Data is taken from Figure S11.

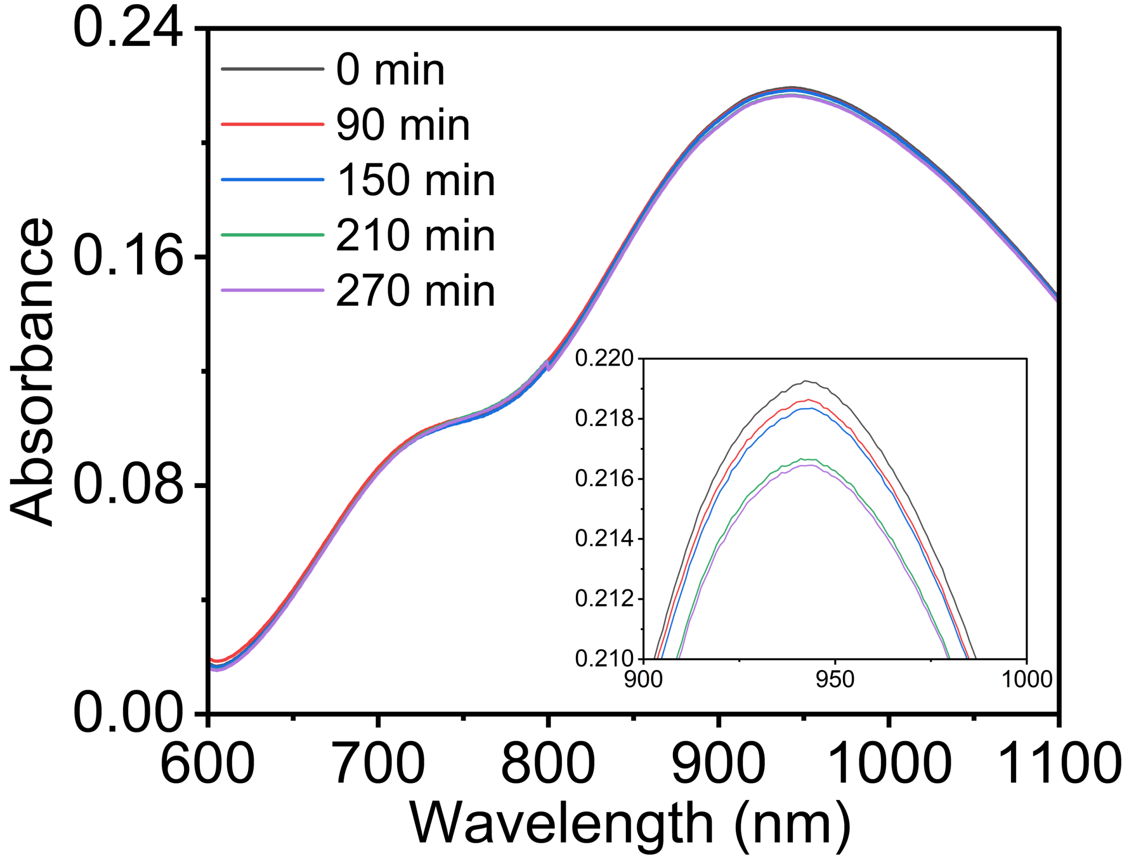

Figure S13. UV-vis analysis of [Cl-Cu^{II}/TPMA] in the presence of RD-6G and excess TPMA in DMSO ([$CuCl_2$]/[TPMA]/[RD-6G] = 1/3/0.1, [$CuCl_2$] = 4.55 mM) under light irradiation (525 nm, intensity: 25 mW/cm^2) at varying times. This model system experiment was conducted in the absence of monomer to isolate the photoreduction kinetics of the copper complex. It shows the reduction of [Cl-Cu^{II}/TPMA] as evidenced by the decrease of the absorption band at ~ 940 nm.

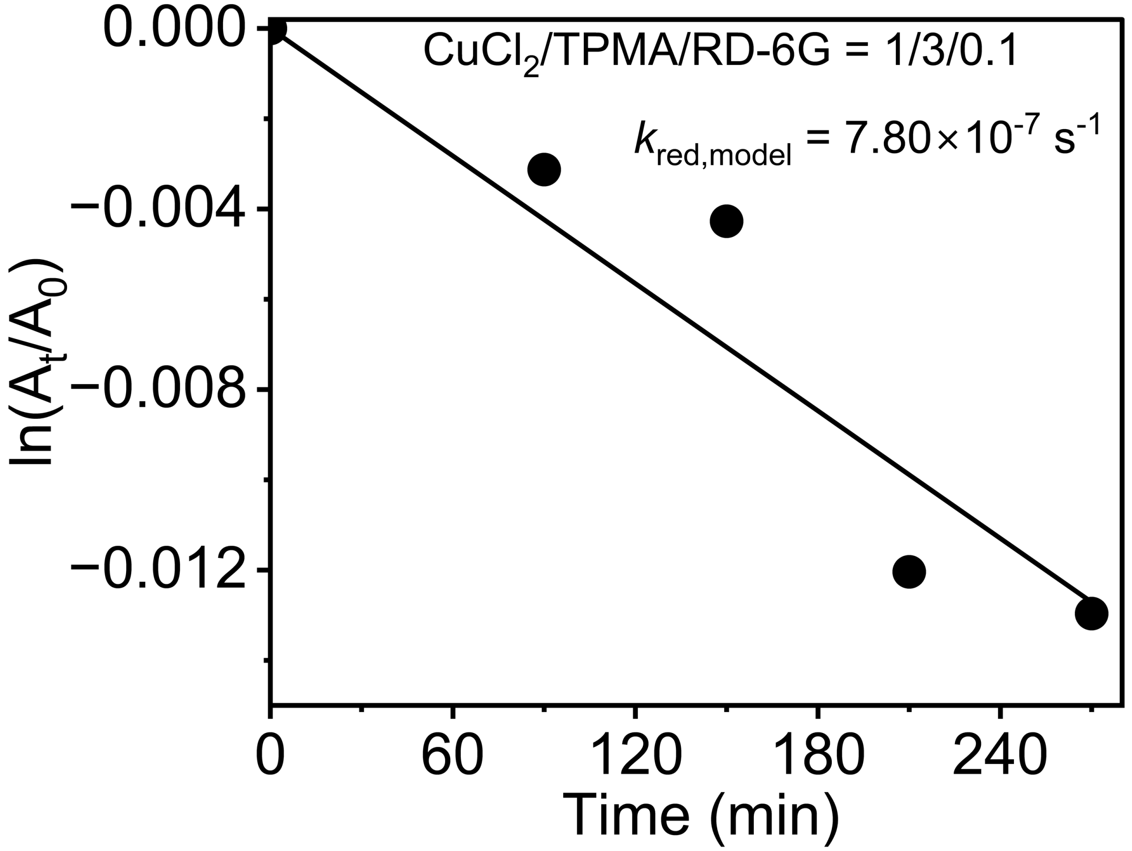

Figure S14. Kinetics of the reduction of [Cl-Cu^{II}/TPMA] in the presence of RD-6G and excess TPMA in DMSO. Data is taken from Figure S17.

Table S1. Summary of electrochemical properties of the copper complexes, thermodynamics and kinetics of their reduction (in the presence of excess ligand and RD-6G under light irradiation, but without monomer), and comparison with polymerization kinetics.

CuX_2	Ligand	$E_{1/2}$ (V)[a]	ΔG_{ox} (eV)[b]	ΔG_{red} (eV)[c]	$k_{red,model}$ (s^{-1})[d]	$k_{p,app}$ (s^{-1})[e]
Br	Me_6TREN	-0.300	-0.790	-0.64	2.3×10^{-3}	3.42×10^{-3} (MA)
Cl	Me_6TREN	-0.413	-0.680	-0.64	9×10^{-4}	2.05×10^{-4} (MA)
Br	TPMA	-0.245	-0.845	-0.20	5.6×10^{-6}	3.25×10^{-5} (MMA)
Cl	TPMA	-0.325	-0.765	-0.20	7.8×10^{-7}	9.80×10^{-6} (MMA)

[a] Half-wave potentials of copper complexes in MeCN at room temperature[2]

[b] ΔG_{ox} represents the thermodynamic driving force for oxidative quenching of ^{1}RD-6G* by X-Cu^{II}/L complexes, calculated as $\Delta G_{ox} = E_{1/2}$ (Cu^{II}/Cu^{I}) – E^{S1}_{red}(RD-6G), E^{S1}_{red}= 1.18 V (vs SCE in acetonitrile)

[c] ΔG_{red} represents the thermodynamic driving force for the reductive quenching of ^{1}RD-6G* by excess ligand, calculated as $\Delta G_{red} = E^{S1}_{ox}$(RD-6G) – E_{ox} (ligand), E^{S1}_{ox} (RD-6G) = -1.09 (vs SCE in acetonitrile).

[d] Reduction rate constants determined from UV-vis kinetic analysis in model systems according to Figure S7-S14. These experiments were conducted in the absence of monomer to isolate the photoreduction kinetics of X-Cu^{II}/L complexes by photoexcited RD-6G and excess ligand

[e] The apparent polymerization rate constants taken from the main text Figures 1a-b, determined from semilogarithmic kinetic plots during actual polymerization with monomer present

UV-vis kinetic studies (Figures S7-S14) were performed in model systems containing X-Cu^{II}/L, RD-6G, and an excess of ligand in DMSO, without a monomer or an alkyl halide initiator. This approach isolates the photoreduction step, specifically the regeneration of Cu^{I}/L activator from X-Cu^{II}/L deactivator by photocatalytic electron transfer, from the ATRP equilibrium present during polymerization.[3] Excluding the monomer removed effects of propagation, termination, and the equilibrium between dormant chains (P_n-X) and

propagating radicals (P_n•), enabling measurement of the intrinsic photoreduction kinetics of each copper complex under controlled conditions.[4]

The reduction rate constants from these model experiments ($k_{red,model}$) correlated with the apparent polymerization rate constants ($k_{p,app}$) observed during MA and MMA polymerizations. This indicates that the photoreduction step is a key kinetic factor in determining overall polymerization rates.[3] Systems with faster photoreduction of X-Cu^{II}/L (higher $k_{red,model}$) generated the Cu^{I}/L activator more quickly, resulting in higher radical concentrations and faster polymerization. This trend is evident when comparing halogens, where Br-based systems exhibit faster model photoreduction kinetics and faster polymerization than Cl-based systems.

However, $k_{red,model}$ values from model systems may differ quantitatively from the effective reduction rate constants ($k_{red,app}$) during actual polymerization, as described in equation 4 of the main text. During polymerization, the photoreduction step is coupled to the ATRP equilibrium, and the polarity of the reaction environment changes as polymerization proceeds.[3] Interactions of copper complexes, competitive coordination, and local concentration differences may also affect *in situ* reduction kinetics.

RD-6G can undergo an oxidative or reductive quenching in its singlet excited state. The redox potential of the singlet excited state of RD-6G (^{1}RD-6G*) are $E^{S1}{}_{ox}$ = -1.09 and $E^{S1}{}_{red}$= 1.18 V vs SCE in acetonitrile[5]

In the oxidative quenching pathway, ^{1}RD-6G* reacts with the X-Cu^{II}/L to generate the activator, Cu^{I}/L. In the reductive quenching mechanism, ^{1}RD-6G* is reduced by the

excess L. The ΔG values of these redox processes were calculated according to the following equation:

$\Delta G = E_{ox} - E_{red}$

Thermodynamically, the negative ΔG values correspond to a spontaneous, very fast diffusion-controlled process. Table S1 shows that there is an agreement between the ΔG values and the kinetics of the reduction rates of the X-Cu^{II}/L determined by UV analyses.

Table S2. Results of ATRP of MA using X-Cu^{II}/Me_6TREN and RD-6G with varying degree of polymerization[a]

Entry	[CuX_2]	DP_T	Time (h)	Conv. (%)[b]	$M_{n,theo}$ ($kg{\cdot}mol^{-1}$)[c]	$M_{n,app}$ ($kg{\cdot}mol^{-1}$)[d]	[d]Đ
1	$CuBr_2$	100	2	94	8.30	8.14	1.12
2	$CuBr_2$	200	2	88	15.5	13.5	1.08
3	$CuBr_2$	400	2	87	30.7	29.2	1.05
4	$CuBr_2$	800	2	83	58.6	68.4	1.05
5	$CuCl_2$	100	4	90	7.9	8.65	1.26
6	$CuCl_2$	200	4	84	14.8	13.7	1.07
7	$CuCl_2$	400	4	75	26.6	26.6	1.04
8	$CuCl_2$	800	4	67	47.6	49.6	1.03

[a] For PMA-Br = [MA]/[EBiB]/[$CuBr_2$]/[Me_6TREN]/[RD-6G] = 100/x/0.005/0.015/0.00001, for PMA-Cl = [MA]/[ECiB]/[$CuCl_2$]/[Me_6TREN]/[RD-6G] = 100/x/0.04/0.12/0.0005, V_{MA} = 1 mL, V_{MA}/V_{DMSO} = 1/1, [MA] = 5.55 M, under Ar (λ ~ 527 nm, intensity: 80 $mW{\cdot}cm^{-2}$) [b] Determined by ^{1}H NMR [c] Determined by ^{1}H NMR $M_{n,theo}$ = ([MA]/[Initiator]) × Conv.× M_{MA}+$M_{Initiator}$ [d] Determined by GPC in THF using poly(methyl methacrylate) standards [e] $Đ = M_w/M_n$ (by GPC)

Table S3. Results of ATRP of MMA using X-Cu^{II}/TPMA and RD-6G with varying degree of polymerization[a]

Entry	[CuX_2]	DP_T	Time (h)	Conv. (%)[b]	$M_{n,theo}$ ($kg{\cdot}mol^{-1}$)[c]	$M_{n,app}$ ($kg{\cdot}mol^{-1}$)[d]	[d]Đ
1	$CuBr_2$	100	24	90	7.95	8.65	1.26
2	$CuBr_2$	200	24	87	17.7	15.9	1.23
3	$CuBr_2$	400	24	93	37.5	31.6	1.32
4	$CuBr_2$	800	24	97	77.9	56.8	1.50
5	$CuCl_2$	100	24	94	9.6	10.7	1.17
6	$CuCl_2$	200	24	89	18.0	18.2	1.13
7	$CuCl_2$	400	24	85	34.2	31.5	1.13
8	$CuCl_2$	800	24	74	59.4	59.3	1.16

[a] For PMMA-Br = [MMA]/[EBPA]/[$CuBr_2$]/[TPMA]/[RD-6G] = 100/x/0.001/0.005/0.001, For PMMA-Cl = [MMA]/[ECPA]/[$CuCl_2$]/[TPMA]/[RD-6G] = 100/x/0.04/0.2/0.004, V_{MMA} = 1mL, V_{MMA}/V_{DMF} = 1/1, [MMA] = 4.70 M, under Ar (λ ~ 527 nm, intensity: 80 $mW{\cdot}cm^{-2}$), t = 24 h, [b] Determined by ^{1}H NMR [c] $M_{n,theo}$ = ([M]/[Initiator]) × Conv.× M_{MMA} + $M_{Initiator}$ [d] Determined by GPC in THF using poly(methyl methacrylate) standards [e] $Đ = M_w/M_n$ (by GPC).

References

(1) Jazani, A. M.; Yilmaz, G.; Baumer, M.; Sobieski, J.; Bernhard, S.; Matyjaszewski, K. Unraveling the Roles of Amines in Atom Transfer Radical Polymerization in the Dark. *JACS* **2025**, *147* (15), 12562–12573. DOI: 10.1021/jacs.4c18496.

(2) Qiu, J.; Matyjaszewski, K.; Thouin, L.; Amatore, C. Cyclic voltammetric studies of copper complexes catalyzing atom transfer radical polymerization. *Macromolecular Chemistry and Physics* **2000**, *201* (14), 1625–1631. DOI: https://doi.org/10.1002/1521-3935(20000901)201:14<1625::AID-MACP1625>3.0.CO;2-9.

(3) Ribelli, T. G.; Konkolewicz, D.; Bernhard, S.; Matyjaszewski, K. How are Radicals (Re)Generated in Photochemical ATRP? *Journal of the American Chemical Society* **2014**, *136* (38), 13303–13312. DOI: 10.1021/ja506379s.

(4) Lorandi, F.; Fantin, M.; Matyjaszewski, K. Atom Transfer Radical Polymerization: A Mechanistic Perspective. *Journal of the American Chemical Society* **2022**, *144* (34), 15413–15430. DOI: 10.1021/jacs.2c05364.

(5) Yasui, S.; Tsujimoto, M.; Itoh, K.; Ohno, A. Quenching of a Photosensitized Dye through Single-Electron Transfer from Trivalent Phosphorus Compounds. *The Journal of Organic Chemistry* **2000**, *65* (15), 4715–4720. DOI: 10.1021/jo000448i.
